# Clients’ experiences and satisfaction with produce prescription programs in California: a qualitative evaluation to inform person-centered and respectful program models

**DOI:** 10.3389/fpubh.2024.1295291

**Published:** 2024-03-20

**Authors:** Elizabeth C. Rhodes, Rafael Pérez-Escamilla, Ngozi Okoli, Amber Hromi-Fiedler, Jaime Foster, John McAndrew, Beatriz Duran-Becerra, Kathleen O’Connor Duffany

**Affiliations:** ^1^Hubert Department of Global Health, Rollins School of Public Health, Emory University, Atlanta, GA, United States; ^2^Emory Global Diabetes Research Center, Emory University, Atlanta, GA, United States; ^3^Department of Social and Behavioral Sciences, Yale School of Public Health, Yale University, New Haven, CT, United States; ^4^Yale-Griffin Prevention Research Center, Derby, CT, United States; ^5^Community Alliance for Research and Engagement, Yale School of Public Health, Yale University, New Haven, CT, United States; ^6^Wholesome Wave, Bridgeport, CT, United States

**Keywords:** person-centered, qualitative research, food security, produce prescription, United States

## Abstract

**Background:**

Produce prescription programs have strong potential to improve food security, fruit and vegetable consumption, and health across the life course. Understanding clients’ experiences and satisfaction with produce prescription programs is critical for evaluating the person-centeredness and quality of these programs. The objectives of this study were to (1) describe client experiences and satisfaction with produce prescription programs, with an emphasis on the extent to which they felt they were treated with respect and dignity, and (2) identify recommendations for improving client experiences.

**Methods:**

We conducted four focus group discussions with clients of produce prescription programs in two Federally Qualified Health Centers in California. We used a modified framework analysis approach and organized participants’ experiences with programs into themes.

**Results:**

Three themes captured participants’ program experiences. First, *respectful produce prescription programming* encompassed interactions with individuals delivering the programs that felt respectful (e.g., program staff showing they cared about participants’ health and offering timely assistance with financial incentives) and disrespectful (e.g., not receiving prompt responses to questions about incentives), as well as aspects of program design perceived to be respectful (e.g., provision of gift cards as financial incentives, which offered privacy when purchasing produce). Second, having *autonomy* to use gift cards to choose their preferred fresh fruits and vegetables was viewed as a positive experience, though participants desired greater autonomy to shop at stores other than the program designated stores. Third, participants frequently discussed *program usability*, with some reporting that joining the programs and using the cards was easy, and others describing difficulties activating cards and using them at stores due to cashiers’ lack of awareness of the programs. Overall, participants were highly satisfied with the programs. To improve client experiences, they recommended increasing privacy (e.g., by educating cashiers on the programs so that clients do not need to explain in public what the card is for) and autonomy (e.g., allowing cards to be used at other chain or local stores).

**Discussion:**

Our findings inform efforts to make produce prescription programs more person-centered and respectful, which in turn may increase program demand, engagement, and impact.

## 1 Introduction

The United States (US) is facing the large and growing threat of food insecurity, defined as a lack of continuous access through socially acceptable means to nutritious and safe foods in the amounts needed for a healthy and active life (1). Disproportionately affecting those living in poverty and low-resource settings, food insecurity drives health inequities through multiple pathways such as poor-quality diets, particularly high consumption of low-cost, energy-dense ultra-processed foods and sugar-sweetened beverages and low intake of water, fruits, vegetables, whole grains, and other healthy foods (2). Produce prescription programs have gained traction as a “Food is Medicine” intervention that can reduce food insecurity (3).

In these programs, health care providers “prescribe” fruits and vegetables at the same time that they provide patients with vouchers or debit cards that can be used at retail locations to purchase produce, and/or provide access to produce at healthcare facilities or by delivering at home (4, 5). Eligibility for participation commonly includes patients who have or are at risk for diet-related non-communicable diseases like type 2 diabetes and cardiovascular disease, and who experience food insecurity (4, 6). Produce prescription programs for pregnant women and children, adolescents, and their caregivers are also underway (7–10). Mounting evidence suggests that produce prescription programs can generate substantial health and economic benefits. Studies have found that these programs increase purchasing and consumption of fruits and vegetables, reduce food insecurity, and improve health indicators such as body mass index, hemoglobin A1C, and diastolic blood pressure (10–19). A microsimulation model estimated that produce prescription programs implemented nationally for US adults with diabetes and food insecurity could save $39.6 billion in health care costs and $4.8 billion in productivity costs (12). From a health perspective, the intervention was highly cost effective, with an incremental cost-effectiveness ratio of $18,100/quality-adjusted life years (12). It was also cost saving from a societal perspective (12).

As produce prescription programs are adapted to new contexts and scaled, making these programs person-centered is important; that is, they should be “respectful of and responsive to individual preferences, needs, and values” (20). It is now widely recognized that the provision of high-quality health services is essential for improving population health, and that a key element of high-quality services is that they are person-centered (7). Indeed, evidence links positive health service experiences with patient satisfaction, service utilization, and improved health outcomes (21).

Evaluating person-centeredness involves understanding both client experiences and satisfaction with programs (22). Client experiences of programs is a process indicator of person-centeredness, while client satisfaction is an outcome of client experiences of programs that reflects the extent to which the services provided meet their needs and expectations (22). Although a growing body of literature has explored client experiences and satisfaction with produce prescription programs (23–30), there is still a need to better understand whether program clients feel they are treated with respect and dignity, a key domain of positive client experiences (22, 31). Furthermore, more input and insights from clients on how produce prescription programs can promote positive experiences can be useful for informing the design and delivery of person-centered and respectful programs.

The two objectives of this qualitative research study were to (1) describe client experiences and satisfaction with produce prescription programs, with an emphasis on their perceptions of the extent to which they felt they were treated with respect and dignity, and (2) identify recommendations for improving client experiences. Our study can inform program co-design and person-centered implementation of produce prescription programs in similar contexts in the US.

## 2 Methods

### 2.1 Study team and reflexivity statement

This research was conducted through an equitable partnership between the Yale-Griffin Centers for Disease Control and Prevention (CDC) Prevention Research Center at Yale School of Public Health (Y-G PRC) and Wholesome Wave, a national organization dedicated to improving health equity by making fruits and vegetables more accessible and affordable. Wholesome Wave funds and supports the implementation of produce prescription programs, and Y-G PRC conducts evaluations of these programs in collaboration with a colleague at Emory University. As part of our collaborative work, we are also leading national efforts to promote person-centered and respectful produce prescription program models. For the present study, staff from Wholesome Wave worked jointly with the study team which included the Y-G PRC team and a colleague from Emory University to develop the research objectives, recruit participants, and develop data collection instruments. The study team members collected and analyzed the data, with Wholesome Wave staff providing contextual information about the produce prescription programs. Together, the authors have rich practice, policy, and/or research experience with food assistance programs, including produce prescription programs. Throughout the research process, the study team practiced reflexivity (32). During team discussions, we acknowledged our subjectivity based on our own life and professional experiences, including our knowledge, expectations, and views about the programs, and reflected on our potential influence on the research process before finalizing the focus group discussion (FGD) guide and reaching consensus on the organization and interpretation of the data (32).

### 2.2 Study design, setting, and population

Using a cross-sectional design, we conducted four FGDs with clients from produce prescription programs at two Federally Qualified Health Centers (FQHCs) in Sacramento, California (hereafter referred to as Center A and Center B). Both centers serve predominately low-income populations with a high prevalence of food insecurity. At Center A, the produce prescription program served two population groups: adult parents or guardians of children receiving pediatrics care; and adults with pre-diabetes or type 2 diabetes receiving primary care and participating in the US Diabetes Prevention Program. At Center B, the produce prescription program served adults with pre-diabetes or type 2 diabetes receiving primary care.

### 2.3 Produce prescription programs

**Figure 1:**
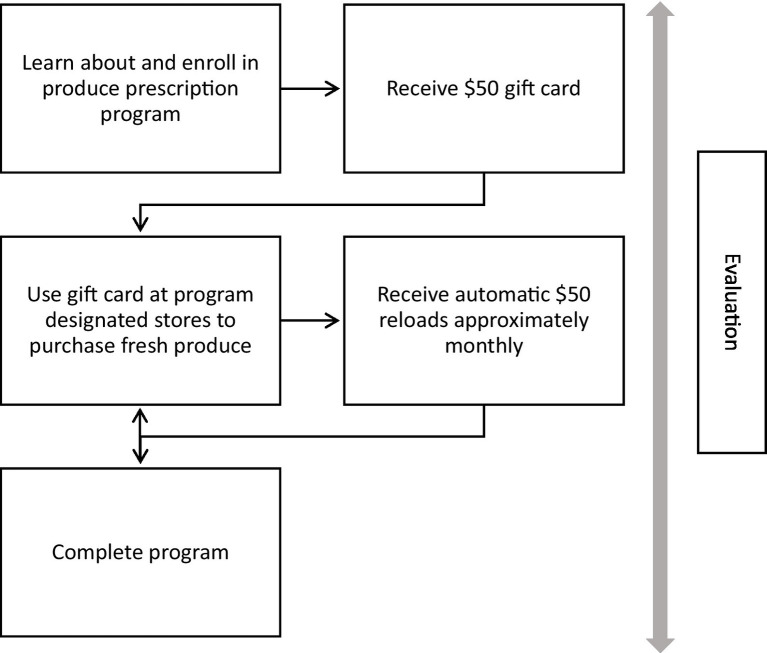
Produce prescription program components.

In collaboration with FQHCs, Wholesome Wave designed and began operating the produce prescription programs in 2021. For clients, there were five main program components: (1) learn about and enroll in the program; (2) receive a financial incentive ($50 gift card) to purchase fresh fruits and vegetables at a designated chain of stores that offers a broad assortment of products and sells food at some but not all locations; (3) use the gift card to purchase fresh produce in person at program designated stores (not online); (4) receive automatic reloads of $50 on the gift card approximately monthly for the duration of the program; and (5) complete program evaluation (e.g., surveys, health assessments) (see Figure 1). The role of FQHC providers was to share program information and enroll individuals in the programs, as well as complete health assessments as part of the evaluation of programs. Wholesome Wave staff were responsible for sending and reloading the gift cards and administering the surveys for program evaluation. Wholesome Wave staff also offered assistance via phone and text when clients had questions about the programs or needed help with the gift cards (e.g., activating the cards). Across programs and target populations, there were variations in the level of assistance provided with the enrollment process and completion of evaluation surveys, as well as differences in how clients received the gift card (i.e., in person or via mail). The duration of the programs ranged from 6 to 8 months. We evaluated clients’ experiences with all components of the programs including the evaluation, since many produce prescription programs have monitoring and evaluation systems in place.

### 2.4 Sampling and recruitment

We used purposive sampling to recruit participants for four FGDs and achieve diversity in our sample with regards to client characteristics (i.e., parents or guardians of pediatric patients, people with prediabetes or diabetes) and preferred language (i.e., English, Spanish). Program clients received text messages from Wholesome Wave about the study, and those who expressed interest were called and/or texted by the study team. In total, the study team contacted 57 clients, reached 25, consented 20, and 17 were available to join an FGD.

### 2.5 Data collection and processing

Qualitative data collection occurred from June to July 2022. Three of the FGDs were conducted with participants who had recently completed the programs, and one FGD was conducted with participants who were active program clients. FGDs were guided by a semi-structured guide, which included a free list activity in which participants were asked open-ended questions that encouraged them to share positive experiences, negative experiences, and recommendations for improving client experiences with each program component, starting with learning about and enrolling in the program and ending with the program evaluation component (Supplementary File 1). A benefit of this FGD guide structure was that the questions followed a logical order for FGD participants, as they mirrored the way in which they progressed through the programs (32). Questions also elicited information about participants’ overall satisfaction and views on programs creating opportunities for feedback from clients. To refine the guide, we held several feedback sessions with the Y-G PRC Community Advisory Group to obtain input from community partners and community members. Once the guide was finalized in English, it was translated into Spanish and checked by a native Spanish speaker.

Each FGD was conducted via Zoom by one trained and experienced moderator and one notetaker, lasted approximately 75 min, and was audio-recorded. The moderator and notetaker for the Spanish FGD were both native Spanish speakers. Immediately following each FGD, the notetaker expanded the notes from the discussion and saved the lists of positive experiences, negative experiences, and recommendations generated during the free list activity. An iterative process of data collection was followed whereby the study team held a debriefing session after each FGD to identify issues in the data to explore in more depth in subsequent FGDs (32). These debriefing sessions were also used to reflect on and discuss the data as they were being collected to identify when data saturation was achieved (i.e., the point in data collection when no more new information is being identified and further data collection become redundant) and thus determine when to stop data collection (32, 33). Each audio recording was then transcribed verbatim by a professional transcription and translation service. The audio recording of the FGD conducted in Spanish was transcribed and translated into English, using a simultaneous translation and transcription approach whereby translation and transcription are conducted simultaneously leading to a single transcript in English (32). Research assistants checked each transcript by listening to the full audio recording while reviewing the transcript and made any necessary revisions to ensure accuracy. A research assistant who is a native Spanish speaker and who moderated the Spanish-language FGD checked the translation of that FGD for accuracy and appropriateness to ensure the translation conveyed the correct meaning (32). To maintain the anonymity of participants, identifying information like participant names was then removed from each transcript. FGD participants were mailed a letter and gift card to thank them for their time.

### 2.6 Data analysis

We used a modified framework analysis approach, which was well-suited to this project given our applied research and practice goals and team that included members with varying levels of qualitative research experience (34, 35). The analysis team included four doctoral-level researchers (ECR, KOD, RPE, JF) and one MPH-level research assistant (NO). Two analysts (ECR, NO) familiarized themselves with the data by reading all four FGD transcripts and then collaboratively and iteratively developed deductive codes from the topics of interest in the FGD guides and components of the programs with the full analysis team. Modifying the framework analysis approach, we also used inductive strategies to identify emergent codes in the data (32, 36). One analyst (NO) applied the coding framework to the FGD transcripts using MAXQDA 2022. This analyst was trained in qualitative research and had a strong understanding of the codes related to the program components as well as other codes given her role in developing the FGD guide, moderating the FGDs, reading the transcripts closely, and contributing to the development of the codebook. A second analyst (ECR) then reviewed the coded transcripts to ensure appropriate application of codes and consistency in the application of the codes across the dataset. Once the data were coded, two analysts (ECR, NO) read the coded segments closely and developed detailed narrative descriptions of the findings for each code, incorporating information from FGD notes and lists generated during the free list activities. These narrative descriptions replaced the tabular summaries of the charting procedures of framework analysis.

To share the findings with key partners, we produced a report of the preliminary findings regarding positive and negative experiences according to each of the program components (Supplementary File 2). For this paper, we conducted an in-depth analysis of participants’ experiences with programs to further organize the findings into meaningful themes to support future program improvement. Members of the analysis team generated themes through a collaborative process that involved grouping data on participants’ experiences with programs into categories informed by concepts from the literature on user experiences of health services, combining these categories into themes, and reaching consensus on final themes (31, 32). Throughout the analysis process, the data analysts returned to the data, re-reading the full transcripts of all four FGDs multiple times to help ensure that the findings were well grounded in the data (32).

## 3 Results

Table 1 presents characteristics of the individuals who participated in the FGDs. Most participants were between 18 and 64 years of age. The majority identified as women (82.4%). Participants identified as Black (29.4%), White (23.5%), Latino (29.4%), and Asian (11.8%). Education levels ranged from less than high school/GED through completion of a bachelor’s degree. Most participants reported using the gift card one to two times a month.

**Table 1:** Characteristics of all focus group discussion participants (*N* = 17).

Characteristics	*N* (%)
Age, categories in years	
18–29	4 (23.5)
30–44	5 (29.4)
45–64	7 (41.2)
65 or over	1 (5.9)
Gender	
Woman	14 (82.4)
Man	3 (17.6)
Non-binary (neither, both, or something else)	0 (0.0)
Race/ethnicity	
Black	5 (29.4)
Asian	2 (11.8)
White	4 (23.5)
Other^*^	5 (29.4)
Preferred not to report	1 (5.9)
Hispanic/Latino	
Yes	5 (29.4)
Education	
Less than high school/GED	2 (11.8)
High school/GED completed	3 (17.6)
Some college or associate’s degree	9 (53.0)
Bachelor’s completed	3 (17.6)
Coursework above a bachelor’s degree	0 (0.0)
Preferred not to report	0 (0.0)
How often did they use the gift card?	
Less than once a month	1 (5.9)
Once a month	8 (47.0)
Once every two weeks	6 (35.3)
Once a week	1 (5.9)
More than once a week	1 (5.9)

^*^All participants who reported their race/ethnicity as “other” reported being of Hispanic/Latino origin.

First, we provide an in-depth description of participants’ program experiences, organized into three themes: respectful produce prescription programming; autonomy to make food choices and select shopping locations; and program usability. We then present findings regarding participants’ satisfaction with the programs, followed by their recommendations for improving client experiences with produce prescription programs.

### 3.1 Client experiences with produce prescription programs

#### 3.1.1 Respectful produce prescription programming

To participants in most FGDs, respectful produce prescription programs should involve both respectful interpersonal interactions with people delivering the program and program elements that are designed to promote feelings of respect. The extent to which participants felt treated with respect and dignity during interpersonal interactions varied. When describing instances of respectful or disrespectful treatment in interpersonal interactions, participants focused on how clinic staff, program staff, and store employees did or did not practice respectful communication and support. They explained that program staff were helpful during the enrollment process, describing staff as “very sweet” in assisting them with activating their gift cards. Participants in some FGDs pointed out that when offering the program, clinic staff were “very respectful in the way they presented it.” For example, clinic staff did not make them feel “ashamed” to use the program and treated them as equals, in contrast to some programs where “people talk down” to them:

*For me, they didn't treat you like you were lower than anybody. They just had a program. They wanted you to try it. They thought it would benefit you because of whatever my conditions were like that to help us to get better. So it was for something, a positive outcome to help me to be a better person, to be healthier and to eat the right kind of things that most likely I wouldn't eat before.* (FGD 1)

During the program, participants felt that program staff practiced respectful communication and support by explaining information about the program, providing updates about reloads, responding to questions, and resolving issues participants encountered with using the cards. Similarly, participants felt respected when program staff were proactive in their communication, such as providing advance notice of delayed reloads:

*I’ve been treated with respect. When I was on the program, … they was going to send the money late on the card, and they texted me ahead of time and tell me that the money was going to be a little bit late.* (FGD 4)

Participants in one FGD felt respected because they had a number to call to check their card balance or ask questions, indicating to them that they had help available if needed. On the other hand, they felt disrespected when they reached out to program staff about card issues or questions about card reloads and did not receive responses:

*When they’re taking us off the program, I think at least, they could tell us that, starting next month you won’t be getting anymore refills, because I’ve been waiting and I text and I text and I ask and no response. So, that’s what I think they fall short. They could have texted or sent us that you’re going to be discontinued off the program starting next month.* (FGD 4)

Participants also shared that they felt respected when clinic staff, program staff, and store employees were patient or made a concerted effort to assist them. For example, participants reported that store employees were patient with them as they learned how to use their gift cards for the first time. They also shared stories of store employees asking other employees how to process the card when they were unsure themselves and helping to call support when the card would not work. They shared similar stories of clinic staff:

*So, when I went in [to the clinic] and I told them about [the program], the lady in the front was just like, oh I’m not sure what program exactly that you’re talking about, but let me go ahead and get you someone that could possibly know what you’re talking about. So she went in the back, got me someone. And then that lady….was the one that was like, oh well I’m surprised that your provider didn’t tell you about it. She apologized that the provider didn’t tell me about it, so she was just like, I’m sorry that you didn’t get the information, but yes, and this is the program that we’re offering. And she was able to give me the steps, I got my card literally that day…So whatever the provider lacked in letting me know about it, they definitely picked it up on the reception end of it.* (FGD 2)

In contrast, participants in one FGD reported encounters with clinic staff who did not listen to their requests for a health assessment, “were not very nice” when they asked for a health assessment, or did not make an effort to complete one, despite it being a part of the program.

Program implementers “showing that they really cared” about participants’ health and well-being was another key reason participants felt like they were treated with respect and dignity during the programs. Participants described that program staff showed genuine interest in their health and demonstrated they cared by offering the program. When program staff addressed card issues, it also indicated to participants that staff were invested in optimizing their health:

*When I first took the card out for the first time, I couldn't activate it, and I had to spend my own money. I was a little frustrated about it at first. So, then I got a hold of the Wholesome people and they were [very passionate]. It was like, ‘Oh no problem. So sorry this happened to you. This is what you do. A, B and C.’ And that and stuff. He goes, ‘If it doesn't work for you, please get back to me.’ And that made me know that the program wasn't giving us a handout like you see in so many programs… I felt that they genuinely were trying to … help us to eat better, help us to be healthier. Their mind was in the right place.* (FGD 1)

A prominent example of a program design element that fostered a sense of respect included program provided gift cards that afforded clients privacy when purchasing produce, particularly in contrast to more conspicuous forms like EBT cards. Participants in two FGDs shared that they liked the privacy offered by the gift cards. For example, one participant commented, “When you go [to the program designated store], just use as a gift card, nobody have to know what you are buying or what you are cooking or what you do not like, it’s all private.” In particular, they appreciated that the card did not indicate that they had a health condition like diabetes. As one participant shared, “[The gift card] does not single you out on what you are using it for, for a medical condition or anything like that. Nobody really knows why you are using the card, what it is.” Participants also explained that the gift card could not be easily identified by others in the store as cards from a food assistance program, which may make clients more willing to use the cards:

*I think that the meaning of they making a gift card also makes it respectful because when you pay with this card, it’s not like an EBT card. So, you’re saying I’m using a gift card to buy these vegetables… some people might feel uncomfortable showing an EBT card at the store. But if you show this card, which is not very common, they’ll be like, ‘Oh, it’s just another gift card. So think that make it more respectful for people to be willing to go to [the store].* (FGD 4)

For participants, another benefit of having cards that others did not know were from a food assistance program was that it prevented them from experiencing poor treatment by store employees, as they had when using benefits from other food assistance programs:

*Sometimes even with the Food Stamp program or the EBT, sometimes cashiers would look at you and talk to you a certain way. But I didn’t experience that with this program. Because I remember one time going to a store, and after I bought my groceries, I was getting ready to pay, the cashier say, “Are you going to pay with EBT?” And so, I pull out cash and it’s like the expression on her face changed. But she just assumed that I’m going to pay with EBT without really asking.* (FGD 1)

However, this privacy was disrupted when participants needed to explain to store cashiers what the card was for and how to use it. To ensure maximum privacy, participants noted that they did have the option of using self-checkout for their purchases. In addition to having privacy while buying produce, participants appreciated that the programs did not monitor their food purchases, commenting “you can buy your favorite fruits and vegetables over and over and you do not get a warning, hey, you already bought these vegetables, or anything like that.”

While completing program evaluation surveys, participants felt respected. For instance, they perceived baseline survey questions about their past eating habits or medical history to be non-intrusive. In the Spanish-language FGD, participants appreciated that surveys were administered in Spanish, which enabled them to fully express themselves. Additionally, in one FGD, a participant felt that program staff cared about them because survey questions went beyond participants’ experiences with the program and inquired about their overall health and well-being:

*To me, it was showing that they really cared. It was not just about the $50 card. It was about your well-being, that they really were concerned about what your mindset was, what you were going through and also your health. So, it wasn’t just about one thing. It was the overall picture, the big picture.* (FGD 1)

#### 3.1.2 Autonomy to make food choices and select shopping locations

Having control over their food choices fostered a sense of autonomy for participants. They expressed appreciation for the autonomy that came with using a gift card, in contrast to receiving a food voucher or box of food. The gift card gave them freedom to choose, unrestricted, both the desired quantity and types of fresh fruits and vegetables. Still, a few participants highlighted that they liked that the gift card could only be used for fruits and vegetables since it promoted healthy eating.

At the same time, they pointed out two elements of the current program design that limited their autonomy. First, the programs did not allow purchases of different types of fruits and vegetables, like frozen produce. Second, the card could only be used at one designated store chain. Participants strongly preferred having more options where they could purchase produce, in part because transportation to stores was a prominent barrier. The cost of gas was a concern for those with a car; for those without a car, getting to the store required finding a ride or using public transportation, which could be difficult. Additionally, since not having a car and/or farther store distance made multiple trips difficult, participants reported having to spend all the card funds in one trip, which they disliked since purchasing fresh fruits and vegetables at one time meant they had to rush to eat or freeze produce before it spoiled:

*The only problem I have with the program is that you can only use it at [program designated store]. I have a grocery store across the street, but I can’t use it across the street. I have to get a ride to [the store]. And if it was for any grocery store, then I could just go by myself and go when I want to. That way, my vegetables don’t spoil by getting them all at once.* (FGD 4)

Participants also discussed that the program designated store was not their usual shopping location. In some cases, they wanted to shop at Asian or Mexican grocery stores. Others reported needing to visit multiple stores because they had to make a separate trip to buy produce at the designated store. Additionally, participants pointed out that some program designated store locations, including some closest to them, did not sell food:

*In most of [the program designated stores], they don’t have food there. So, I had to find the ones that had food, and they were pretty much out of my area. So, I’d have to have somebody take me, drive me, to go there. So, I wish it would have it at other stores…You know, stores that we actually go to.* (FGD 1)

Of the program designated stores that sold food, the selection of fresh fruits and vegetables was perceived by some participants to be “limited” and low quality, which meant participants searched for other stores or did not use the cards:

*I wish [the gift card] wasn’t just specifically at [the program designated store]. Because [the program designated store] doesn’t always have the best fruit or vegetable and I just feel like, and don’t get me wrong sometime I saved up my card almost a hundred dollars just because they didn’t have fruits or vegetables for us or at least where it was more like good ones, like a good shipment instead of oh this is bad, just throw it out anyways and sell it.* (FGD 2)

Furthermore, there was consensus that fresh fruits and vegetables at the program designated stores were more expensive than produce at other stores. This was not preferable for participants who wanted to save money.

#### 3.1.3 Program usability

Participants frequently discussed positive experiences with regards to the ease of use of the programs, but also pointed out usability issues that negatively affected their experiences and engagement in the programs. Many participants found the enrollment process to be quick, hassle-free, and “very straightforward.” Even participants who described themselves as less tech-savvy or “computer illiterate” found the online sign-up process easy to follow. Some participants received their cards the same day or within a few days after signing up for the program, while others experienced long wait times between signing up and receiving their gift cards.

Most participants reported that activating and using the gift cards at the checkout counter or self-checkout was easy. For some participants, once they “got the hang of” using the cards, it was “very convenient,” since all they had to do was swipe the card at the machine at checkout. For some participants, however, the gift cards were not as easy to use. Difficulties with using the gift cards occurred when store cashiers did not know how to process payments using the cards, and when cards would not swipe at card machines. These difficulties frustrated participants, especially when they purchased the items with money they had not budgeted for fresh produce:

*When I first got the card, [the program designated store] wouldn’t accept it at first…I said, “Well, the money’s on there.” And I didn’t know how explain to them how to take it off. I didn’t know. I think they had did it with credit, and I was trying to get them to take it off as a gift card or something…And when I got the groceries that time, I had to pay out of pocket to get it. So, I wasn’t too happy about that part.* (FGD 1)

Additionally, some participants found the initial information they received about the program (e.g., instructions on how to use the card, guidelines on foods that could and could not be purchased with the card) easy to follow, while others reported that this information was unclear, leading to confusion over which foods (e.g., pre-cut vegetables, frozen vegetables) could be purchased with gift cards. They described instances when they thought produce like frozen vegetables and pre-packaged salads could be purchased with gift cards, and upon learning at checkout that they could not be, then had to use their own money to pay for these items.

Participants liked that the reloads occurred automatically and that confirmations of the reloads were sent via text message. Moreover, they liked having their cards reloaded automatically around the same time each month because it allowed them to plan their grocery trips in advance. Similarly, they appreciated receiving text messages with their remaining card balance. These aspects of the program were perceived as “convenient” and gave them one less thing to worry about. In the Spanish-language FGD, participants reported receiving text messages in English, making it difficult for them to understand information about card reloads. Participants also discussed differing experiences with regards to being notified about future reloads. Additional challenges arose when gift cards were lost or stolen requiring a replacement, cards were not automatically reloaded due to lack of use when their intention was to save funds to buy produce in bulk, or cards were not reloaded on a regular schedule making it difficult to “budget things” and plan grocery trips, especially when relying on others for transportation to the store. Lastly, some participants did not have clarity on the length of time they could participate in the program and when it would end. In one FGD, a few participants felt that the notification of their final program month came with little warning, making them feel like the program came to a sudden end. Nevertheless, others appreciated having notice that the program was ending, even if they received this notice during the last month of the program.

Regarding the program evaluation, most participants found the surveys to not take too long to complete, and those who completed health assessments liked that they could go to the health centers for the assessments at times that were convenient for them. However, some participants reported that not all clinic staff were aware that the program evaluation involved health assessments, which created confusion and negative encounters for participants during clinic visits and meant clients had to explain the health assessment to clinic staff:

*I need to say this about the health assessment. In the email that we receive about going to get our blood drawn also says to ask a nurse to weigh the client or the patient and get their blood pressure and height. And I did this multiple times. And I don’t know what was going on with [the clinic staff], but they weren’t really aware of this program, or at least not everybody was aware. So, every time I went to get my blood drawn, I had to explain the whole process. It’s not about my doctor asking me to get my A1C check, it’s about this program, blah, blah, blah.* (FGD 4)

### 3.2 Satisfaction with produce prescription programs

Participants reported that they were highly satisfied with the programs. Important drivers of their satisfaction were having an extra $50 each month for purchasing fresh fruits and vegetables as well as the healthier eating and health benefits that resulted. For example, one participant who spontaneously rated the program shared:

*I would say 9 out of 10. And I was satisfied because…I thought it was very beneficial for my kids, instead of waiting on a paycheck coming in, we have a gift card that could help you for vegetables and fruits for the time being.* (FGD 2)

Many participants appreciated having extra money dedicated to fresh fruits and vegetables, particularly during the COVID-19 pandemic and periods of inflation that led to increases in the cost of fruits and vegetables. Using the gift cards and having additional funds each month also motivated participants to eat and try healthier foods since the money could only be used to buy fresh fruits and vegetables. Not only did the gift cards allow participants to buy foods that they usually did not buy, but it also allowed them to afford more expensive produce that they usually would not purchase due to high costs. Participants with children saw improvements in their children’s eating habits and felt motivated to eat healthier and try new foods. Additionally, participants viewed the extra $50 per month as money saved to pay for non-food expenses like health care costs or emergencies. However, some participants pointed out that $50 was not always enough to cover the high cost of fresh fruits and vegetables nor did it fully offset the high cost of travel associated with traveling to the store by car or public transportation. Overall, participants expressed gratitude for having the opportunity to participate in the programs and hoped that the programs would be able to reach and benefit more individuals and families in the future.

Despite having some negative experiences with the programs, such as having to travel far distances to the grocery store, not knowing what foods were covered under the programs, and having interactions with clinic staff during health assessments that felt “disrespectful,” participants still described high levels of satisfaction. They explained that it is difficult to complain when the program offers free gift cards for fruits and vegetables:

*I definitely was satisfied with the program. I think anytime you get something, I don't want to say for free, but any help that you can get, how can you complain about that? And I did notice my four year old, she's pretty picky, she definitely found more fruits and vegetables that she likes. I feel like we had more of a variety for her to try.* (FGD 2)

### 3.3 Recommendations for improving client experiences

Participants shared key recommendations across all themes related to client experiences (see Table 2). To make produce prescription programs more *respectful*, participants recommended that programs ensure respectful communication – for example, by ensuring that program staff are responsive to clients’ requests for assistance with gift cards and that text messages are sent in clients’ preferred language. Participants also suggested increasing the inclusivity of programs by increasing the accessibility and reach of programs so that more people could benefit. Participants frequently discussed their preference for greater *autonomy* within programs, including the ability to purchase additional types of produce, use gift cards at more stores, and use funds any time. Finally, participants offered numerous recommendations for improving the *usability of programs*. They desired more information about program parameters such as types of produce that can be purchased, as well as better communication between program and clinic staff. They also recommended ways to make programs more user-friendly, from providing training for cashiers to more efficient processes such as making it easier to obtain replacement gift cards and reach program staff with inquiries. Furthermore, all participants were supportive of programs creating greater opportunities for them to voice program issues and concerns and provide feedback to inform iterative program improvements. For example, one participant commented that program implementers “could improve the program by [incorporating perspectives from] the people who have experience using the program” and be “made aware of what little issues might be there and they can be ironed out,” which would “make the program a lot…smoother.”

**Table 2:** Participants’ recommendations for improving client experiences (*n* = 4 FGDs).

Theme	Recommendation	Reported examples of specific actions
Respectful produce prescription programming	Ensure respectful communication	Ensure that program staff are responsive to clients when they ask for assistance with card issues Ensure that messages are sent to clients in their preferred language
	Increase the accessibility of programs	Provide an online purchase and delivery option to facilitate program participation among individuals with disabilities
	Increase the reach of programs	Engage additional sub-populations (e.g., older adults, individuals enrolled in SNAP, individuals not enrolled in SNAP)
Autonomy to make food choices and select shopping locations	Design programs that provide clients with greater autonomy	Expand the type of produce (e.g., frozen fruits and vegetables) that can be purchased with gift cards Allow gift cards to be used at more stores (e.g., local ethnic grocery stores, stores closer to where clients live) Do not restrict the timing of when funds can be used (e.g., have rollover of funds each month)
Program usability	Ensure clients understand program parameters	Make sure all clients understand the gift card’s restrictions, especially with regards to which foods can be purchased Provide advanced notification for the last reload
	Improve communication between program and clinic staff	Ensure all clinic staff are aware of the program and its requirements, such as health assessments
	Make programs user-friendly	Provide training for store cashiers to increase awareness of the program and knowledge of how to use gift cards Create an easier process for replacing gift cards Have a phone number that clients can call to talk to program staff directly about card issues Have reloads occur at the same time each month to help clients better plan grocery trips Have an easier way to check the balance on gift cards Simplify the health assessment process (e.g., by having designated days or times that assessments are completed, use pre-existing health information in the electronic health record)
	Offer more opportunities for client input	Get feedback from participants about their experiences with gift cards, including to make sure that they did not have issues activating the cards and that they understand any restrictions (e.g., types of foods that can be purchased)

FGDs, focus group discussions; SNAP, Supplemental Nutrition Assistance Program.

## 4 Discussion

This qualitative study evaluated client experiences and satisfaction with produce prescription programs and identified opportunities to make these programs more person-centered and respectful moving forward. Most participants viewed respectful produce prescription programs as encompassing both respect in interpersonal interactions with individuals delivering the program (e.g., being treated well, experiencing timely communication) and program design elements (e.g., provision of gift cards that offer privacy when purchasing produce). Participants liked having the ability to choose their own fresh produce, but they preferred to not be limited to shopping only at program designated stores. Participants spoke at length about both positive and negative experiences with program usability, highlighting the many ways that ease of use of the programs strongly shaped their experiences. All participants were highly satisfied with the produce prescription programs, despite having some negative experiences. Finally, participants offered numerous recommendations for optimizing the client experience, such as increasing privacy (e.g., by educating store employees on the program so that clients do not need to explain in public what the card is for), expanding autonomy (e.g., by allowing cards to be used at more stores), and addressing issues related to program usability (e.g., creating a simpler process for replacing lost or stolen gift cards).

Many of the positive and negative program experiences as well as the recommendations for program improvement that were described by study participants are consistent with existing literature. Participants felt that people delivering the programs treated them with respect and dignity when they showed that they cared about participants’ health and well-being. In a qualitative research study, Schlosser and colleagues found that participants in a produce prescription program felt cared for by providers when they took time to discuss healthy eating (24). Participants in our study also felt respected when store employees made an effort to assist them in using their gift cards during checkout. Previous qualitative studies of produce prescription programs documented that participants reported having positive interactions with farmers’ market vendors when they were friendly and helped participants use their vouchers (25, 26).

Participants liked that the gift cards gave them autonomy to choose their own fresh fruits and vegetables. Similarly, Saxe-Custack and colleagues found that most caregivers participating in a pediatric produce prescription program preferred selecting their own fruits and vegetables at farmers’ markets over receiving vendor-prepared produce bags (26). Notably, our study participants desired greater autonomy, particularly more options for the types of produce that could be purchased (e.g., frozen produce) and the stores where gift cards could be used. Similarly, previous studies have found that participants want incentives that can be used at multiple locations (27, 28). In our study, one primary reason participants wanted expanded options for where to purchase produce was that they faced challenges with transportation, a commonly reported barrier to redemption (23, 24, 37, 38). They also wanted to shop at Asian or Mexican grocery stores as well as stores with greater variety and higher quality produce, noting that in some cases they did not use the cards because of limited or low-quality produce. These findings are consistent with an evaluation of a produce prescription program in which participants reported that they had faced challenges with accessing high-quality fruits and vegetables before the COVID-19 pandemic and these challenges worsened during the pandemic (39). A study conducted by Lyonnais and colleagues found that participants reported that available locations where vouchers could be redeemed did not have the foods they were looking for and that this was a barrier to voucher redemption (29). Esquivel and colleagues documented similar concerns among participants in a pediatric produce prescription program (25). For instance, participants wanted to use vouchers for foods beyond fruits and vegetables (25). Their ability to select the fruits and vegetables they wanted was also diminished when using an online market due to the limited variety of produce available and the inability to personally hand pick the produce of highest quality (25, 27).

Participants in this study frequently discussed ease of use of the programs, which has been an area commonly reported upon in studies evaluating produce prescription programs. Making vouchers easy to use is a widely known facilitator to program engagement, while difficulties with voucher use such as expiration dates and vouchers that require all funds be used at one time have been documented as barriers to engagement, including use of the full voucher amount (25, 40). Moreover, previous studies found that participants want and suggest that vouchers and incentives not have an expiration date or that the expiration date be extended (27, 28). In a qualitative study on perceptions of a produce prescription program designed for Supplemental Nutrition Assistance Program (SNAP) clients, the most frequently reported reason participants did not use their incentives was difficulties with using them at checkout, which led to embarrassment and feelings of stigmatization by cashiers and other customers (28).

By exploring the experiences and perspectives of produce prescription program clients, this study expands the literature on the experiences and views of clients and adds an explicit focus on respectful programming. It also complements a small but growing body of research focused on the experiences and perspectives of health care providers implementing produce prescription programs (11, 24, 37). For example, a recent study conducted by Stotz and colleagues found that health care providers were strongly satisfied with produce prescription programs and described their positive effects on patient care (11). Similarly, we found that participants were highly satisfied with the produce prescription programs, especially given the health benefits of the programs.

Importantly, previous research has found that high satisfaction with health services is common in low-resource settings, even when services are of poor quality (31). While satisfaction is influenced by the quality of health services, it is also affected by other factors such as immediate outcomes of services, gratitude, and an individual’s needs, expectations, and values (22, 31). In our study, participants indicated that their satisfaction was driven largely by their perceived benefits of the program for themselves and their families, including increased ability to purchase and consume produce and improved health. These perceived benefits have been documented in many previous qualitative and mixed methods studies evaluating clients’ perspectives of produce prescription programs in a wide variety of contexts and populations. Consistent with existing literature, participants in our study also expressed strong appreciation for the programs and shared many positive experiences, including positive interactions with program staff, clinic staff, and store employees (23–26, 28, 30, 41). At the same time, they indicated that they had low expectations regarding how they were treated by cashiers. They reported previous instances of being mistreated when using EBT cards, expressed acceptance that they may experience mistreatment while using produce prescription program gift cards, and explained that they focused on the benefits of these programs rather than mistreatment by cashiers. These findings underscore that client satisfaction as a measure should be used carefully, as high satisfaction may be due, in part, to low expectations as well as other factors (22, 31). Any mistreatment violates people’s fundamental right to be treated with respect and dignity (22). Evaluation of client experience can provide insights on the quality of produce prescription programs including respectful treatment, while satisfaction can shed light on the extent to which these programs are responsive to clients’ expectations (22).

### 4.1 Strengths and limitations

This study had several strengths. By co-developing the FGD guide with a Community Advisory Group, we created questions and a free list activity that promoted active discussion in which FGD participants probed and explained themselves to each other. This high level of interaction elicited a wide range of views on program experiences and opportunities for improvement and generated rich, nuanced data on areas with strong consensus and differing views among participants. Conducting this research through an equitable partnership enhanced our ability to produce results of high relevance and utility for healthcare systems and organizations implementing produce prescription programs.

This study also had limitations. First, three FGDs had fewer than the target number of six to eight participants due to challenges with scheduling at times that work for most but not all interested clients and clients agreeing to participate but then being unable to attend the FGDs. Despite the small size of these FGDs, there was still active group interaction wherein participants built on each other’s comments and debated and justified issues. As such, the FGDs still brought out valuable insights and a variety of perspectives. Second, we conducted one FGD with Spanish speakers. It is possible that new issues would have been identified if we had conducted additional FGDs with Spanish speakers. However, it is important to point out that we did not identify major differences in views and experiences between English-speaking and Spanish-speaking participants. Third, as explained in the methods section, the FGDs were conducted via Zoom, which may have made it more challenging to build rapport compared with in-person FGDs. The detailed information and stories that participants shared, including both positive and negative experiences, indicated that participants felt comfortable to share openly and honestly. Fourth, the data were coded by one analyst and inter-coder reliability was therefore not assessed. Recognizing the importance of ensuring the analyst who coded the data applied the codes appropriately and consistently across the four transcripts, another analyst checked segments of the coded transcripts. Notably, no issues with the application of codes were identified.

Finally, there are a variety of ways to design produce prescription programs. For example, the two programs evaluated in this study offered $50 gift cards to purchase fresh produce in person at designated stores, while some programs offer vouchers or debit cards of varying amounts that can be used to purchase both fresh and frozen produce in stores or online. As such, findings regarding clients’ experiences and satisfaction with various aspects of the programs evaluated in this study may not be directly transferable to all produce prescription programs in the US or other countries, but the methods used to elicit them are applicable as they were based on pragmatic and rigorous approaches to collecting and analyzing qualitative data. Process and impact evaluations of clients’ experiences and satisfaction with programs that are designed differently are warranted.

### 4.2 Lessons and opportunities for research and practice

In 2022, the Biden-Harris Administration National Strategy on Hunger, Nutrition, and Health highlighted policy and practice activities for expanding and increasing utilization of produce prescriptions in various types of government-sponsored healthcare programs (42). National agencies like the Indian Health Service, Veterans Health Administration, Centers for Medicare and Medicaid Services, US Department of Agriculture (USDA), and CDC, as well as state and local level government agencies, community-based organizations, and health systems are pushing this agenda forward in states across the country. As pointed out by Downer and colleagues, there is a need for greater investment in studies in the US using rigorous study designs that can produce high-quality evidence on the effects of produce prescription programs on food security, diet quality, and cardiometabolic health outcomes (4). As research on produce prescription programs expands, it will be important to include a focus on client experiences with programs, including the extent to which they feel treated with respect and dignity (1). Qualitative research is well suited to exploring client experiences with programs and understanding how, why, and in what context produce prescription programs promote positive experiences, including respectful treatment (1). A validated measure to quantitatively assess client experiences and respectful treatment is also sorely needed.

Optimizing person-centered and respectful program delivery requires tailoring, which our study indicates could be achieved through co-design, an approach wherein members of the community and community partners actively collaborate to develop social innovations (43). A major advantage of this approach is that it centers the voices and perspectives of community members and community partners (43). Program and research-funding agencies like USDA, donors, and others could encourage and offer funds for co-design of produce prescription programs.

## 5 Conclusion

This study sheds light on clients’ experiences and satisfaction with produce prescription programs in California and identifies their specific recommendations for making programs more person-centered – insights that can inform programming among similar populations in the US. This focus will be vital to upholding people’s fundamental right to be treated with respect and for facilitating optimal use of produce prescription vouchers or debit cards, and thereby improving the effectiveness of programs.

## Data availability statement

The datasets presented in this article are not readily available because they include focus group discussion transcripts. Requests to access the datasets should be directed to ER, elizabeth.rhodes@emory.edu.

## Ethics statement

All the methods were carried out in accordance with the relevant guidelines and regulations. The project was reviewed and deemed exempt from further review by the Yale University Institutional Review Board. We chose to obtain verbal informed consent to participate in an audio-recorded focus group discussion from all study participants.

## Author contributions

ER: Writing – original draft, Writing – review & editing, Conceptualization, Investigation, Formal analysis. RP-E: Writing – review & editing, Conceptualization, Investigation, Formal analysis. NO: Writing – original draft, Writing – review & editing, Investigation, Formal analysis. AH-F: Writing – review & editing. JF: Formal analysis, Writing – review & editing. JM: Writing – review & editing. BD-B: Writing – review & editing, Investigation. KD: Writing – review & editing, Conceptualization, Investigation, Formal analysis.
