# Supplementary material for: Clients’ experiences and satisfaction with produce prescription programs in California: a qualitative evaluation to inform person-centered and respectful program models
Source: Front Public Health. 2024 Mar 20;12:1295291. doi: 10.3389/fpubh.2024.1295291 (PMC10990041; doi:10.3389/fpubh.2024.1295291)
Supplement: Supplementary file 1 [file Table_1.docx]

**Supplementary File 1**

**Semi-structured focus group discussion guide questions**

| **Topic** | **Focus group discussion questions** |
| --- | --- |
| Learning about programs^1^ | - How did you learn about the program? |
| Motivation for joining programs^1^ | - Why did you decide to join the program? |
| What it means to be treated with respect | - In your opinion, what does it mean to be treated with respect and dignity by people who you interact with during the program? |
| Experiences of produce prescription programs and recommendations for improving client experiences | In a free list activity, participants were asked the following questions that encouraged them to share positive and negative experiences with each program component^2^, as well as recommendations for improving client experiences of the component:   - What are things that make [PROGRAM COMPONENT] a positive experience, if any? - What are things that make [PROGRAM COMPONENT] a negative experience, if any? - What changes could Wholesome Wave make so that people have a more positive experience of [PROGRAM COMPONENT], if any? |
| Satisfaction | - Overall, how satisfied were you with the program? Why? |
| Recommendations for improving client experiences | - What are the most important changes Wholesome Wave could make so that people who participate in the program have a positive experience, if any? - Wholesome Wave would like to provide more opportunities for program participants to share their ideas and feedback about the program. What do you think about this idea? |

^1^Opening questions were included in the focus group discussion guide to help moderators build rapport with participants so that they would feel comfortable sharing their experiences and perspectives.

^2^Program components included: learn about and enroll in program; receive $50 gift card; use gift card at program designated store to purchase fresh produce; receive automatic $50 reloads approximately monthly; and program evaluation.
