# Supplementary material for: Clients’ experiences and satisfaction with produce prescription programs in California: a qualitative evaluation to inform person-centered and respectful program models
Source: Front Public Health. 2024 Mar 20;12:1295291. doi: 10.3389/fpubh.2024.1295291 (PMC10990041; doi:10.3389/fpubh.2024.1295291)
Supplement: Supplementary file 2 [file Table_2.DOCX]

**Supplementary File 2**

**Table: Positive and negative experiences reported by clients participating in produce prescription programs in Sacramento, California. Findings from focus group discussions (n=4) organized by program component.**

| **Program component** | **Positive experiences** | **Negative experiences** |
| --- | --- | --- |
| Learn about and enroll in program | - Program staff were helpful during enrollment process - Clinic staff were “very respectful in the way they presented [the program],” did not make participants feel “ashamed” to use it, and did not treat them like they “were lower than anybody” - Program staff showed genuine interest in participants’ health and demonstrated they cared by offering the program - Quick and straightforward enrollment process | - Received multiple text messages from staff encouraging them to join the program despite having enrolled |
| Receive gift card | - Received cards shortly after signing up for programs - Program staff were “very sweet” in assisting with card activation | - Long wait times for gift cards after enrolling in program |
| Use gift card to purchase produce | - Program staff responded to questions and resolved issues participants encountered with using gift cards - Clinic staff, program staff, and store employees were patient (e.g., when clients were first learning how to use their gift cards) and made an effort to assist them - Programs provided a number to call to check card balance or ask questions - Gift cards offered privacy at grocery stores - Programs did not monitor food purchases - Using gift cards offered control over selection of produce - Information about the program was clear (e.g., instructions on how to use the card, foods that could be purchased) - Gift cards could be used easily at the check-out counter or self-check-out - Text messages with remaining card balance were sent to participants | - Did not receive responses when reached out to program staff about card issue or questions about card reloads - Privacy of gift cards was disrupted when participants needed to explain to store cashiers what the card was for and how to use it - Not using funds because of barriers to in-person shopping (e.g., disability, not having a car, high-risk for severe COVID-19 symptoms) - Programs did not allow purchases of non-fresh fruits and vegetables (e.g., frozen produce) - Card could only be used at one store chain - Unclear program information (e.g., which foods could be purchased, duration of program) - Did not receive text messages in preferred language (i.e., Spanish) - Poor communication between program staff and clinic staff, which meant that in some cases clients had to explain the program to clinic staff - Difficulties with using gift cards (e.g., when store cashiers did not know how to process payments using cards) - Lost or stolen gift cards required having to get replacement cards |
| Receive automatic fund reloads | - Reloads occurred automatically around the same time each month and confirmations of reloads were sent via text messages - Program staff were proactive in their communication (e.g., provided advance notice of delayed reloads) | - Cards were not reloaded on a regular schedule, making it difficult to “budget things” and plan grocery store trips - Cards were not automatically reloaded due to lack of use (e.g., when participant intended to save funds to buy produce in bulk) |
| Program evaluation | - Survey questions that went beyond participants’ experiences with the programs and inquired about their overall health and well-being indicated to participants that program staff cared about them - Baseline survey questions were non-intrusive - Surveys were administered in Spanish - Surveys did not take too long to complete - Health assessments could be done at times convenient for participants - End-of-program surveys gave participants an opportunity to share their opinions about the programs | - Participants in one FGD felt clinic staff did not listen to their requests for a health assessment, “were not very nice” when they asked for health assessments, or did not make an effort to complete them |
